# Real value prediction of protein solvent accessibility using enhanced PSSM features

**DOI:** 10.1186/1471-2105-9-S12-S12

**Published:** 2008-12-12

**Authors:** Darby Tien-Hao Chang, Hsuan-Yu Huang, Yu-Tang Syu, Chih-Peng Wu

**Affiliations:** 1 Department of Electrical Engineering, National Cheng Kung University, Tainan, 70101, Taiwan, R.O.C; 2 Department of Computer Science and Information Engineering, National Taiwan University, Taipei, 10617, Taiwan, R.O.C

## Abstract

**Background:**

Prediction of protein solvent accessibility, also called accessible surface area (ASA) prediction, is an important step for tertiary structure prediction directly from one-dimensional sequences. Traditionally, predicting solvent accessibility is regarded as either a two- (exposed or buried) or three-state (exposed, intermediate or buried) classification problem. However, the states of solvent accessibility are not well-defined in real protein structures. Thus, a number of methods have been developed to directly predict the real value ASA based on evolutionary information such as position specific scoring matrix (PSSM).

**Results:**

This study enhances the PSSM-based features for real value ASA prediction by considering the physicochemical properties and solvent propensities of amino acid types. We propose a systematic method for identifying residue groups with respect to protein solvent accessibility. The amino acid columns in the PSSM profile that belong to a certain residue group are merged to generate novel features. Finally, support vector regression (SVR) is adopted to construct a real value ASA predictor. Experimental results demonstrate that the features produced by the proposed selection process are informative for ASA prediction.

**Conclusion:**

Experimental results based on a widely used benchmark reveal that the proposed method performs best among several of existing packages for performing ASA prediction. Furthermore, the feature selection mechanism incorporated in this study can be applied to other regression problems using the PSSM. The program and data are available from the authors upon request.

## Background

Predicting protein tertiary structures directly from one-dimensional sequences remains a challenging problem [1]. The studies of solvent accessibility have shown that the process of protein folding is driven to maximal compactness by solvent aversion of some residues [2]. Therefore, solvent accessibility is considered as a crucial factor in protein folding and prediction of protein solvent accessibility, also called accessible surface area (ASA) prediction, is an important step in tertiary structure prediction [3].

Traditionally, predicting solvent accessibility is regarded as either a two- (exposed or buried) or three-state (exposed, intermediate or buried) classification problem. Various machine learning methods have been adopted, including neural networks [4-11], Bayesian statistics [12], logistic functions [13], information theory [14-16] and support vector machines (SVMs) [17-19]. Among these machine learning methods, neural networks were the first technique used in predicting protein solvent accessibility and are still extensively adopted in recent works. In addition, SVMs were also effective for ASA prediction. Several features were used to train these machine learning methods, such as local residue composition [4,5], probability profiles [20] and position specific scoring matrix (PSSM) [21].

However, subdividing residues into states requires selection of specific ASA values as thresholds, which are not well-defined in real protein structures. The applicability of state ASA predictors is thus limited as their performance is highly dependent on arbitrarily defined thresholds [22,23]. Ahmad *et al*. addressed this problem and developed a method, RVP-net, to predict the real values of relative solvent accessibility (RSA) [22]. The RVP-net used the local amino acid composition to train a neural network and yielded a mean absolute error (MAE) of 18.0–19.5%. Yuan and Huang [23] also used the local amino acid composition and adopted support vector regression (SVR) (the regression version of SVM) to achieve an MAE of 17.0–18.5%. Adamczak *et al*. [24] used the PSSM to train neural networks, which yielded an MAE of 15.3–15.8%. After Adamczak's work, the PSSM was widely used for real value ASA prediction with some success. Wang *et al*. [25] proposed a real value ASA predictor with an MAE of 16.2–16.4% by combining the PSSM with multiple linear regression. Garg *et al*. [26] combined the PSSM and secondary structure information with neural networks to predict RSA with an MAE of 15.2–15.9%. Nguyen and Rajapakse [27] used the PSSM to construct a two-stage SVR, which further improved the MAE to 14.9–15.7%.

Table 1 summarizes the recent developments in predicting real value ASA. Neural networks and SVRs were extensively adopted and outperformed other machine learning methods. However, the difference among alternative regression tools is relatively small in comparison with the introduction of the PSSM (Table 1). This reveals the importance of the feature set in real value ASA prediction. This study focuses on the feature set and proposes a systematic process to enhance PSSM-based features.

**Table 1:** The recent developments, in chronological order, for real value ASA prediction

Work	Regression tool	Description of features	MAE (%)^1^
Ahmad *et al*., 2003	NN^2^	Amino acid composition	18.8
Yuan and Huang, 2004	SVR^3^	Amino acid composition	18.5
Adamczak *et al*., 2004	NN	PSSM^4^	15.3–15.8^5^
Wang *et al*., 2005	MLR^6^	Amino acid composition, PSSM and sequence length	16.2
Garg *et al*., 2005	NN	PSSM and secondary structure information	15.9
Nguyen and Rajapakse, 2006	Two-stage SVR	PSSM	15.7

^1^Mean absolute error of real RSA values. All the methods were evaluated with a three-fold cross-validation on the Barton dataset, except Adamczak *et al*. used their own dataset. ^2^Neural network. ^3^Support vector regression. ^4^Position specific scoring matrix. ^5^The MAEs reported in this work were evaluated on a different dataset to other works. ^6^Multiple linear regression.

For a protein sequence, the PSSM describes the likelihood of a particular residue substitution at a specific position based on evolutionary information [21]. The basic idea of the enhanced PSSM is to merge similar residues into groups and use group likelihood to generate novel features [28,29]. The likelihood of a residue group is obtained by accumulating the PSSM columns of member residues into a single column. A feature selection mechanism is proposed to identify the residue groups appropriate for real value ASA prediction. Based on the proposed selection mechanism, grouped residues are guaranteed to have similar physicochemical properties and solvent propensities. Finally, the features produced by selected residue groups are combined with a two-stage SVR to construct a real value ASA predictor.

The present method is compared with five real value ASA predictors using a widely used benchmark. In addition, the predicted ASA values are transformed to ASA states for comparison with seven state ASA predictors. Experimental results demonstrate that the features produced by the proposed selection process are informative for ASA prediction. Moreover, the feature selection mechanism incorporated in this study can be applied to other regression problems using the PSSM.

## Results and discussion

### Datasets

This study collects three independent datasets, Barton, Carugo and Manesh, from previous works for evaluating alternative ASA predictors. Additionally, two small datasets, SMA1 and SMA2, are created for the feature selection mechanism by sampling the Barton dataset. Table 2 lists the detailed statistics for these datasets.

**Table 2:** Summary of the datasets employed in this study

Dataset	# of chains	# of residues	Mean of RSA (%)	Standard deviation of RSA (%)
Barton	500	83448	28.9	28.1
set1	166	26274	28.4	27.8
set2	167	26720	28.7	28.1
set3	167	30454	29.6	28.3

Carugo	338	82178	29.9	28.4
set1	113	28871	29.3	28.4
set2	113	27354	29.9	28.4
set3	112	25953	30.5	28.3

Manesh	215	50682	28.5	27.3
set1	72	18770	27.5	26.9
set2	72	15264	29.2	27.4
set3	71	16648	28.9	27.6

SMA1^1^	42	6632	27.6	27.5
SMA2^2^	42	7680	30.9	28.3

^1^This is a subset of the Barton set1. ^2^This is a subset of the Barton set3.

The Barton dataset, prepared by Cuff and Barton in 2000 [7], includes 502 non-homologous protein chains with <25% pairwise-sequence identity. According to previous work [22,23,27], this dataset was divided into three subsets with equal protein chains for cross-validation. These three subsets were used for training, testing, and validation data, which resulted in six evaluation combinations. The performances of the six combinations were averaged as overall performance. The second dataset, Carugo, was prepared by Carugo in 2000 [15], and includes 338 non-homologous monomeric proteins with <25% pairwise-sequence identity. The third dataset, Manesh, was prepared by Manesh *et al*. in 2001 [16], and has 215 non-homologous protein chains with <25% pairwise-sequence identity. These two datasets, Carugo and Manesh, were also divided into three subsets of equal size for cross-validation.

The three evaluation datasets – Barton, Carugo and Manesh – are used to evaluate the present method and to compare alternative ASA predictors. Moreover, the proposed feature selection mechanism requires two datasets. To prevent overfitting, this work uses only a small number of samples from the evaluation subsets with the worst prediction performance in previous work. The worst prediction performance implies that the selected subsets are more distinct than other subset combinations. Consequently, two small datasets, SMA1 and SMA2, are constructed by randomly selecting 42 protein chains from set1 and set3 of the Barton dataset, respectively. Both small datasets account for ~1/4 of the original set from which they are extracted.

The real values of ASA in Barton and Carugo were determined by the Dictionary of Protein Secondary Structure (DSSP) program [30], whereas the values in Manesh were determined by the Analytical Surface Calculation (ASC) program [31] based on the suggested van der Waals radii by Ooi *et al*. [32]. The RSA value of a residue was then computed by dividing the real ASA value by that observed in the extended Ala-X-Ala conformation of the residue. In this study, RSA is used as the main measure for evaluating real value ASA predictors.

### Evaluation measures

Two widely used measures for real value ASA prediction are adopted in this study to evaluate existing ASA predictors. The first measure, mean absolute error (MAE), is defined as follows:

$$\text{MAE}=\frac{\sum_{\text{for each residue}}\left|RSA_{predicted}-RSA_{observed}\right|}{n},$$

where *n *is the total number of residues to be predicted, and MAE is the absolute difference between predicted and observed (from experiments) RSA values. The second measure is Pearson's correlation coefficient (CC), which is defined as follows:

$$\text{CC}=\frac{1}{n-1}\cdot \sum_{\text{for each residue}}\left(\frac{X-\bar{X}}{s_{X}}\right)\left(\frac{Y-\bar{Y}}{s_{Y}}\right),$$

where *n *is the total number of residues to predict; *X *and *Y *are the predicted and observed RSA value of each residue, respectively; $\bar{X}$ and $\bar{Y}$ are the average of predicted and observed RSA values of all residues, respectively; *S*_*X *_and *S*_*Y *_are the standard deviation (calculated using *n*-1 in the denominator) of predicted and observed RSA values of all residues, respectively; CC is the ratio of the covariance between the predicted and observed RSA values to the product of the standard deviations of the predicted and observed RSA values.

### Feature selection

This study enhances PSSM-based features by considering the physicochemical properties and solvent propensities of amino acid types. The concepts of using the property- and propensity-based PSSM (called PSSMP) have been used in some classification problems. Shimizu *et al*. [28] first introduced the concept of the property-based PSSM by grouping residues belonging to a certain physicochemical property. Such residue groups exploit evolutionary information of a particular property at a specific position. The construction details of PSSM and PSSMP features can be found in the Methods section.

However, considering only the physicochemical property to identify residue groups generates an important question: Do all amino acids in a property group contribute consistently in various bioinformatics problems? Hence, Su *et al*. [29] proposed that physicochemical groups can be further divided into sub-groups according to residue propensities for order/disorder to predict protein disorder regions. For example, the property *Small *(V, C, A, G, D, N, S, T and P) was divided into *Small *with order propensity (V, C, N and T) and *Small *with disorder propensity (A, G, D, S and P). Such residue groups consider class propensities and can generate novel PSSM-based features for different problems.

Real ASA prediction, unlike order/disorder classification, lacks a well-defined threshold for measuring solvent propensities of amino acids. Thus, this study develops a novel iterative selection process that identifies the residue groups appropriate for real value ASA prediction without defining a propensity threshold. This process uses a physicochemical property (Table 3) as the initial residue group and removes a member residue with the smallest or largest solvent propensity in each round, until prediction performance cannot be improved (see the Methods section for details). Starting from these properties ensures that grouped residues have similar physicochemical properties. Moreover, removing residues from those with extreme propensities indicates that the remaining residues have similar propensities.

**Table 3:** Conventional physicochemical properties

Property	I	L	V	C	A	G	M	F	Y	W	H	K	R	E	Q	D	N	S	T	P
*Hydrophobic*	Y	Y	Y	Y	Y	Y	Y	Y	Y	Y	Y	Y							Y	
*Polar*									Y	Y	Y	Y	Y	Y	Y	Y	Y	Y	Y	
*Small*			Y	Y	Y	Y										Y	Y	Y	Y	Y
*Aliphatic*	Y	Y	Y																	
*Aromatic*								Y	Y	Y	Y									
*Positive*											Y	Y	Y							
*Negative*														Y		Y				
*Proline*																				Y
*Charged*											Y	Y	Y	Y		Y				
*Tiny*				Y	Y	Y												Y		

This study compares prediction performance to that of the original PSSM and identifies five residue groups with improved performance (Table 4). Finally, all possible combinations of the five groups are evaluated. Care has been taken to prevent the inclusion of *Polar*_*sel *_and *Charged*_*sel *_in a group combination – *Charged*_*sel *_is a subset of *Polar*_*sel*_. The combination with the best prediction performance is the pair composed of *Charged*_*sel *_and *Tiny*_*sel*_. The final feature set with two selected properties is named PSSM-2SP, and is used as the feature set in the present method. The whole feature selection process is based on the two small datasets; that is, the prediction performances of all residue groups and group combinations are obtained using SMA1 to predict SMA2.

**Table 4:** The selected properties with improved performance than the original PSSM

Property	Residue group	Removed residues
*Polar*_*sel*_	KRQDN	YWHEST
*Small*_*sel*_	GDNSTP	VCA
*Negative*^1^	ED	--
*Charged*_*sel*_	KD	HRE
*Tiny*_*sel*_	AG	CS

^1^No amino acid type is removed from the property *Negative *in the iterative selection process.

### Two-stage regression

Following the design by Nguyen and Rajapakse [27], this study adopts two cascading regressions to predict real ASA values. In the first stage, this study uses PSSM-2SP as the feature set, which encodes the level of conservation at a position and the properties of substituted residues. A drawback of this feature set is that it lacks ASA information of neighbor residues. Thus, a second regression is included to account for the contextual information of neighboring solvent accessibility.

The second regression uses the output of the first regression as an estimation of neighboring solvent accessibility. The *i*-th residue in a protein sequence is represented as a 2*w*+1 dimensional vector **v **= (*a*_*i*-*h*_, *t*_*i*-*h*_, *a*_*i*-*h*+1_, *t*_*i*-*h*+1_,..., *a*_*i*_, *t*_*i*_,..., *a*_*i*+*h*_, *t*_*i*+*h*_, *l*), where *a*_*i *_is the predicted RSA value of the *i*-th residue in the first regression, *t*_*i *_is the terminal flag as either 1 (a null/terminal residue) or 0 (otherwise), *l *is the sequence length and *w *= 2*h*+1 is window size.

The SVR (see the Methods section for details) is used as the regression tool for both stages in the present method. For a test protein sequence, this study encodes the residues with PSSM-2SP and invokes the first SVR to obtain the first-stage RSA values. These RSA values are then used to encode residues for the second SVR. The RSA values predicted by the second SVR are the final output of the proposed ASA predictor. This study adopts the widely used LIBSVM package (version 2.86) for SVR implementation [33]. All required parameters are determined using SMA1 to predict SMA2. These parameters are constant in all 18 evaluation combinations of the three evaluation datasets. Table 5 shows these parameters.

**Table 5:** Parameters used in this study

Parameter	Value
In the first regression	
SVR kernel	Gaussian
C	2^-1^
γ	2^-7^
ε	2^-6^
Window size	11

In the second regression	
SVR kernel	Gaussian
C	2^3^
γ	2^0^
ε	2^-8^
Window size	3

### Performance on evaluation datasets

The performance of the proposed method is compared to five real value ASA predictors (Table 6). The predictors for comparison are the neural network method developed by Ahmad *et al*. [22], the single-stage SVR developed by Yuan and Huang [23], multiple linear regression developed by Wang *et al*. [25], multiple neural networks developed by Garg *et al*. [26] and the two-stage SVR developed by Nguyen and Rajapakse [27]. All predictors included the Barton dataset as one of the evaluation datasets (Table 6). Although some variants exist in the prediction pipeline (e.g., Wang *et al*. used five-fold cross-validation, Garg *et al*. used seven-fold cross-validation and all other predictors used three-fold cross-validation), the performance on the Barton dataset is still a good benchmark for measuring the effectiveness of these predictors. For the Barton dataset, the MAE and CC of the proposed method are 14.8% and 0.68, respectively, both of which are better than those of the compared predictors.

**Table 6:** Comparison of the present method and five real value ASA predictors on the Barton, Carugo, and Manesh datasets

	Barton		Carugo		Manesh	
			
Method	MAE (%)^1^	CC^2^	MAE (%)	CC	MAE (%)	CC
Ahmad *et al*.	18.8	0.48	19.0	0.48	18.0	0.50
Yuan and Huang	18.5	0.52	--^3^	--	--	--
Wang *et al*.	16.2	0.64	--	--	--	--
Garg *et al*.	15.9	0.65	--	--	15.2	0.67
Nguyen and Rajapakse	15.7	0.66	15.7	0.67	14.9	0.68
**Our method**	**14.8**	**0.68**	**14.8**	**0.69**	**14.2**	**0.69**

Wang *et al*. applied five-fold cross-validation on Barton dataset. Garg *et al*. applied seven-fold cross-validation on Barton dataset and five-fold cross-valuation on Manesh dataset. All other results were obtained by three-fold cross-validation. ^1^Mean absolute error. ^2^Pearson's correlation coefficient. ^3^Indicates that the corresponding result was not available from the literature.

However, the construction of the proposed ASA predictor (which included PSSM-2SP generation and parameter determination) is based on SMA1 and SMA2, which are part of the Barton dataset. Thus, the results from the Carugo and Manesh datasets are helpful in investigating the overfitting effects during the construction process. The improvements to the two datasets by the proposed method are analogous to the improvement to the Barton dataset, suggesting that the overfitting effects of using SMA1 and SMA2 are negligible (Table 6).

Furthermore, the predicted RSA values using the proposed method are transformed into binary ASA states (buried and exposed) for comparison with state ASA predictors. The predictors for comparison are PHDacc [5], Jnet [7], the information theory approach developed by Manesh *et al*. [16], NETASA [10], the probability profile method developed by Gianese *et al*. [20], the two-stage SVM [19] and two-stage SVR [27]. The two-stage SVR approach is also a real value ASA predictor, the results of which were transformed into binary ASA states. Table 7 shows a comparison of existing state ASA predictors. In this experiment, a set of 30 proteins from the Manesh dataset is used as the training set, and the remaining 185 proteins of the Manesh dataset are used as the test set. The proposed method achieves the best accuracy for most thresholds, except at 5% and 10% thresholds (Table 7). Nevertheless, the proposed method still yields an accuracy rate >80% at 5% and 10% thresholds. These experimental results show that the present ASA predictor can classify the buried/exposed state of residues.

**Table 7:** Comparison of the present method and seven state ASA predictors on the Manesh dataset

Method\Threshold (%)	5	9	10	16	20	25	36	50	60	70	80	90
Rost and Sander	--^1^	74.6	--	75.0	--	--	--	--	--	--	--	--
Cuff and Barton	79.0	--	--	--	--	75.0	--	--	--	--	--	--
Manesh *et al*.	--	75.9	--	75.5	--	74.4	74.1	--	--	--	--	--
Ahmad *et al*.	74.6	--	71.2	--	--	70.3	--	75.9	--	--	--	--
Gianese *et al*.	75.7	--	73.4	--	--	71.6	--	76.2	--	--	--	--
Nguyen and Rajapakse												
(Two-stage SVM)^2^	**82.9**	--	**81.0**	--	78.6	78.1	--	79.1	83.4	--	--	--
Nguyen and Rajapakse												
(Two-stage SVR)^3^	81.1	78.7	78.5	77.9	77.6	77.3	76.9	79.5	84.3	89.9	95.0	97.5
Our method	80.9	**80.2**	80.1	**79.4**	**78.7**	**78.5**	**78.4**	**80.8**	**85.3**	**90.7**	**95.0**	**97.8**

This table reports the accuracy (%) of alternative methods based on the training set of 30 proteins from the Manesh dataset to predict the remaining 185 proteins of Manesh. ^1^Indicates that the corresponding result was not available from the literature. ^2^Nguyen and Rajapakse proposed a two-stage SVM approach in 2005 which treats solvent accessibility as a classification problem [19], ^3^and then proposed a two-stage SVR approach in 2006 which treats solvent accessibility as a regression problem [27].

### Prediction performance vs. amino acid type

This study develops a systematic process to identify appropriate residue groups for ASA prediction. However, some amino acid types are not included in the *Charged*_*sel *_and *Tiny*_*sel *_properties. This analysis investigates if the proposed PSSM-2SP improves these amino acid types. Table 8 compares the prediction performance for 20 amino acid types with and without the *Charged*_*sel *_and *Tiny*_*sel *_information. Table 8 reveals some important facts in current real value ASA prediction, such as amino acids that are more hydrophobic (I, L, V and C) are better predicted than those less hydrophobic (E, D, N and S). These MAE differences among amino acid types concur with and have been discussed in previous works [25,27]. Here, this study focuses on improving PSSM-2SP over the PSSM. The PSSM-2SP improves ≥ 0.7% MAE for most amino acid types, although the *Charged*_*sel *_and *Tiny*_*sel *_properties include only A, G, K and D (Table 8). This can be explained by the multiple sequence alignment in constructing the PSSM-2SP. Namely, a non-A, -G, -K and -D residue is still affected by the *Charged*_*sel *_and *Tiny*_*sel *_properties when some of its homology sequences have A, G, K or D residues within the corresponding window.

**Table 8:** Comparison of PSSM and PSSM-2SP on the Barton dataset in terms of amino acid types

		MAE (%)		
		
Amino acid type	Occurence (%)	PSSM	PSSM-2SP	Improvement
I	5.5	9.7	8.7	1.0
L	8.5	10.7	9.8	0.9
V	6.9	10.6	9.6	1.0
C	0.9	9.8	8.9	0.9
A*	8.7	14.1	13.3	0.9
G*	7.8	19.8	19.5	0.4
M	2.0	12.3	11.3	0.9
F	3.9	11.2	10.2	1.0
Y	3.6	13.3	13.0	0.3
W	1.5	12.4	11.8	0.6
H	2.2	15.5	15.1	0.4
K*	5.9	17.1	15.8	1.3
R	4.5	17.7	17.0	0.7
E	6.0	18.9	17.8	1.1
Q	3.7	18.1	17.2	0.9
D*	5.9	20.1	19.2	0.8
N	4.7	20.4	19.6	0.8
S	6.2	19.0	18.3	0.7
T	6.0	16.9	16.0	0.9
P	4.7	18.2	17.4	0.8

## Conclusion

There is an enormous gap between the number of protein structures and the huge number of protein sequences. Thus, predicting protein structures directly from amino acid sequences remains one of the most important problems in life science. The PSSM generated by PSI-BLAST is a useful feature set for sequence-based methods in various bioinformatics problems. This study proposes a novel feature selection mechanism that enhances the PSSM-based features for real value ASA prediction. Based on the selected PSSM-2SP features, this study adopts two cascading SVRs to construct an ASA predictor. The performance of the proposed method is compared with that of five real value ASA predictors and seven state ASA predictors. Experimental results show that the proposed predictor performs best in evaluating datasets. It can predict real ASA values with an MAE of 14.2–14.8% and predict state ASA with an accuracy of 78.4–97.8%. These experimental results demonstrate that the selected features are informative for ASA prediction. Another contribution of this study is the proposed systematic process for generating novel PSSM-based features for regression problems. This is achieved by shrinking the initial physicochemical property from residues with extreme propensities. The feature selection mechanism in this study can be applied to other regression problems using the PSSM.

## Methods

This study adopts an iterative selection process to determine which residues should be grouped together to generate novel features for real value ASA prediction. In each round, this process generates new residue groups, transforms the dataset into a vector representation according to the residue groups, and evaluates the residue groups by performing SVR on the transformed dataset. Evaluation results are used for construction of residue groups in the next round. This section first describes the workflow of the proposed iterative selection process, and then the details of constructing the feature vector and SVR algorithm.

### The proposed iterative selection process

**Figure 1:**
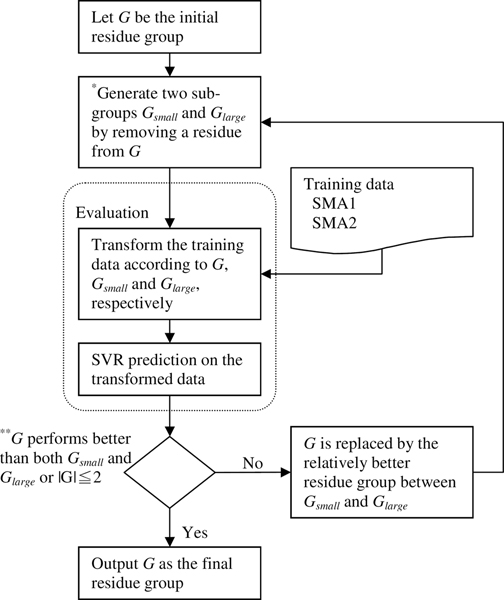
**Workflow of the proposed iterative selection process.** *G_*small *_is generated by removing the residue with the largest average RSA from *G*, and *G*_*large *_is generated by removing the residue with the smallest average RSA from *G*. **The performance of each residue group is measured according to the MAE delivered by SVR. |*G*| is the size of *G*.

**Figure 2:**
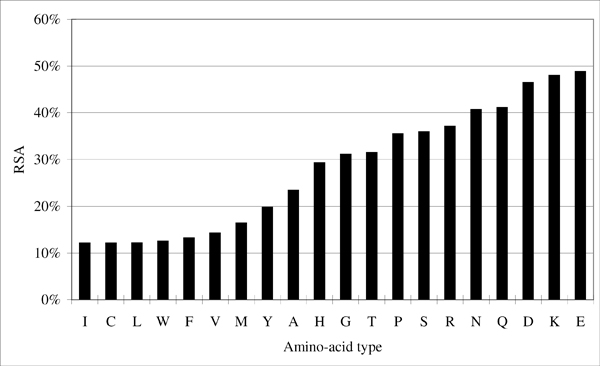
The average RSA value of each amino acid type in the SMA1 and SMA2 datasets.

Figure 1 shows the workflow of the selection process. This iterative selection starts from an initial residue group *G*. In this implementation, nine of the ten physicochemical properties (Table 3) are used as initial groups (the property *Proline *is not used since it includes only an amino acid type). Staring from these properties ensures that residues in the final residue group have similar physicochemical properties. The next step is to generate two sub-groups, *G*_*small *_and *G*_*large*_, from *G*. Suppose that *G *has *n *amino acid types, *G*_*small *_then contains the smallest *n*-1 amino acid types of G in terms of solvent propensity. The solvent propensity of an amino acid type is estimated by averaging the RSA values of all residues of that amino acid type in the SMA1 and SMA2 datasets. Figure 2 shows the RSA averages obtained by examining the residues in the SMA1 and SMA2 datasets. Similarly, *G*_*large *_contains the largest *n*-1 amino acid types of G in terms of solvent propensity.

The three residue groups, *G*, *G*_*small *_and *G*_*large*_, are then evaluated by using SMA1 to predict SMA2. The evaluation step is divided into two sub-steps as described in the following two subsections. If *G *is the best residue group of the three residue groups during evaluation, then the whole selection process is done; otherwise, *G *is replaced by the relatively better residue group between *G*_*small *_and *G*_*large *_in the evaluation step and the next round is started.

One of the most distinct features of this iterative selection process compared to conventional backward selection is that only two sub-groups are considered in each round. There are two reasons for this modification. First, residues in the final residue group are guaranteed to have similar solvent propensities by removing amino acid types from those with extreme propensities. The second advantage is respect to the computational concern. Conventional backward selection generates *n *sub-groups for a group with *n *elements and results in a time complexity of *O*(*N*^2^), where *N *is the size of the initial residue group. The modification in this study reduces time complexity to *O*(*N*).

### Encode residues as feature vectors

The first-stage of the proposed ASA predictor follows the practice of using PSSM-based features to encode residues. This sub-section first describes the construction of the original PSSM, and then that of the PSSMP according to a given residue group *G*. For a protein sequence, construction of the PSSM is achieved by first invoking the PSI-BLAST program [21] to the non-redundant (NR) database obtained from the NCBI, where low-complexity and transmembrane regions and coil-coil segments are removed as suggested by Jones [34]. The settings for PSI-BLAST in this study, including the cutting E-value threshold (*e*) of 10^-3^, multi-pass inclusion E-value threshold (*h*) of 2 × 10^-3^, and iteration count of 3, follow the suggestions of a previous study [35].

The PSSM profile generated by PSI-BLAST consists of the likelihood of a particular residue substitution at a specific position. These likelihood values are rescaled to [0,1] using the following logistic function [36]:

$$x^{\prime }=\frac{1}{1+\exp(-x)},$$

where *x *is the raw value in the PSSM profile and *x*' is the value corresponding to *x *after rescaling. Each position of a protein sequence is represented by a 21-dimensional vector where 20 elements take the likelihood values of 20 amino acid types from the rescaled PSSM profile; the last element is a terminal flag as most PSSM-based methods have introduced [27,29]. Finally, the feature vector based on the original PSSM for a residue comprises a window of positions. For example, the *i*-th residue in a protein sequence is represented as a *w *× 21 dimensional vector, includes the positions *i*-*h*, *i*-*h*+1,..., *i*,..., *i*+*h *of that sequence, where the window size is *w *= 2*h*+1.

**Figure 3:**
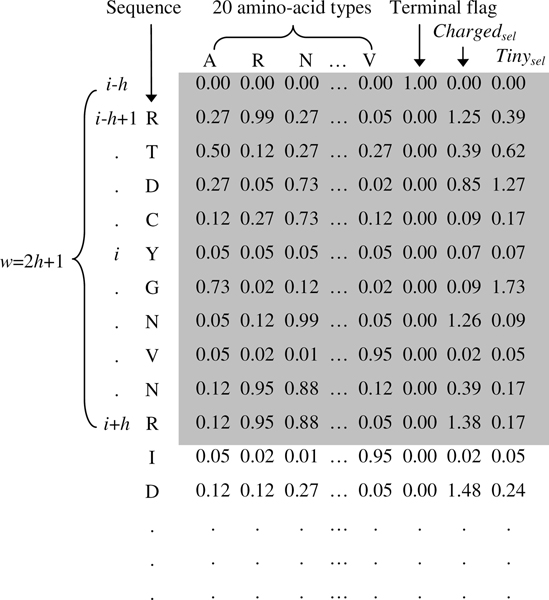
**An example of encoding a residue to its corresponding feature vector**. We encode the fifth residue (*i *= 5) of a protein (PDB ID: 154L) with window size 11 (*w *= 11 and *h *= 5). In this example, a position of the protein sequence is represented by a 23-dimensional vector (20 amino acid values, a terminal flag and two group values). The first row is a pseudo terminal residue where only the terminal flag is 1 and all other 22 values are zero. Finally, the *i*-th residue is encoded with its neighboring positions to form a 253-dimensional feature vector.

After constructing the PSSM profile, the PSSMP profile according to residue group *G *can be generated easily by accumulating the PSSM profile values of residues in *G *to enlarge the profile by one dimension. That is, a one-group PSSMP feature set results in 21 likelihood values at a specific position, where 20 elements are the same as in the original PSSM, and the last value is the accumulated value. This is slightly different from the procedure used by Su and coworkers, which discards likelihood values of residues in *G *and forms a condensed PSSMP [29]. The resulting PSSMP profile is then rescaled to [0,1], added with a terminal flag and then formatted into the vector representation with a window size *w*. Consequently, a residue based on an *n*-group PSSMP is represented as a *w *× (21+*n*) dimensional feature vector. Figure 3 shows an example of encoding a residue to its corresponding feature vector.

### Support vector regression (SVR)

Regression is a technique used for estimating an unknown continuous-valued function based on a set of samples consisting of a dependent variable (response variable) with one or more independent variables (explanatory variables). In real value ASA prediction, each sample (*i.e.*, each residue) is represented by a feature vector, **v**, and an associated RSA value, *y*. Each element in **v **is an independent variable, and *y *is the dependent variable. The SVR is a kernel regression technique that constructs a model based on support vectors. This model expresses *y *as a function of **v **with several parameters:

$$y=b+\sum_{s_{i}\text{ is a support vector}}w_{i}K(v,s_{i}),$$

where *K*() is the kernel function, and *b *and *w*_*i *_are numerical parameters determined by minimizing the prediction error on training samples. A training instance, **s**_*i*_, is selected as a support vector when the associated weight *w*_*i *_exceeds a user-specified threshold, *C*. In addition, SVR introduces the following two criteria to reduce the risk of overfitting when minimizing prediction error: 1) a user-specified parameter, *ε*, defines a tube around the regression function in which errors are ignored; and 2) maximizing the flatness of the regression function. The problem is to find the support vectors and determine parameters *b *and *w*_*i*_, which can be solved by constrained quadratic optimization [37]. The LIBSVM package (version 2.86) [33] is used for SVR implementation in this study. Table 5 lists the values of these user-defined parameters.

## Competing interests

The authors declare that they have no competing interests.

## Authors' contributions

Author DTHC designed the methodology and conceived of this study. HYH, YTS and CPW designed the experiments and performed all calculations and analyses. All authors have read and approved this manuscript.
